# Living on Three Time Scales: The Dynamics of Plasma Cell and Antibody Populations Illustrated for Hepatitis A Virus

**DOI:** 10.1371/journal.pcbi.1002418

**Published:** 2012-03-01

**Authors:** Mathieu Andraud, Olivier Lejeune, Jammbe Z. Musoro, Benson Ogunjimi, Philippe Beutels, Niel Hens

**Affiliations:** 1 Centre for Health Economics Research and Modelling of Infectious Diseases (CHERMID), Vaccine & Infectious Disease Institute (VAXINFECTIO), University of Antwerp, Antwerp, Belgium; 2 The SYMBIOS Center, Division of Mathematics, University of Dundee, Dundee, United Kingdom; 3 Interuniversity Institute of Biostatistics and Statistical Bioinformatics, Hasselt University, Diepenbeek, Belgium; 4 Academic Medical Center, University of Amsterdam, Amsterdam, The Netherlands; Imperial College London, United Kingdom

## Abstract

Understanding the mechanisms involved in long-term persistence of humoral immunity after natural infection or vaccination is challenging and crucial for further research in immunology, vaccine development as well as health policy. Long-lived plasma cells, which have recently been shown to reside in survival niches in the bone marrow, are instrumental in the process of immunity induction and persistence. We developed a mathematical model, assuming two antibody-secreting cell subpopulations (short- and long-lived plasma cells), to analyze the antibody kinetics after HAV-vaccination using data from two long-term follow-up studies. Model parameters were estimated through a hierarchical nonlinear mixed-effects model analysis. Long-term individual predictions were derived from the individual empirical parameters and were used to estimate the mean time to immunity waning. We show that three life spans are essential to explain the observed antibody kinetics: that of the antibodies (around one month), the short-lived plasma cells (several months) and the long-lived plasma cells (decades). Although our model is a simplified representation of the actual mechanisms that govern individual immune responses, the level of agreement between long-term individual predictions and observed kinetics is reassuringly close. The quantitative assessment of the time scales over which plasma cells and antibodies live and interact provides a basis for further quantitative research on immunology, with direct consequences for understanding the epidemiology of infectious diseases, and for timing serum sampling in clinical trials of vaccines.

## Author Summary

Recent studies evidenced the existence of long-lived plasma-cells which could play a major role in the long-term persistence of antibodies after infection or vaccination. A mathematical model, accounting for two plasma-cells populations (short and long-lived), was developed to analyze data from two long-term follow-up studies in patients vaccinated with hepatitis A inactivated vaccines. Parameter estimates confirmed the importance of three time scales to explain the decay of antibody levels: the antibodies lifespan (around one month), the short-lived plasma cells lifespan (several months) and the long-lived plasma cells lifespan (decades). This study also highlighted the need of more frequent observations during the first year post-vaccination to estimate accurately the different parameters governing the long-term antibody dynamics.

## Introduction

The human adaptive immune response relies on a complex combination of cellular and humoral immunity, mediated by T- and B-lymphocytes. Although vaccination aims to activate both cellular and humoral immunity, vaccine induced immunity is typically evaluated by means of the antibody titer, secreted by B-lymphocytes [1]. After encountering antigens, B-cells are stimulated to proliferate and/or differentiate into memory B-cells and plasma cells (PC). Memory B-cells permit a faster and more effective immune response upon further exposures to the antigens, whereas PC are the main antibody-secreting cells (ASC). Different antibody isotopes are present in human sera (IgM, IgA and IgG). They each have relatively limited half-lives, with a maximum of 17.5–26.0 days for Immunoglobulin G (IgG), which represent about 75% of the antibody isotopes in humans [2], [3], [4]. Nonetheless, exposure to common viral and vaccine antigens has been shown to induce a long-term humoral immune response, which illustrates that improving our understanding of the mechanisms involved in the production and persistence of antibodies remains a (relatively rarely explored) topic of fundamental scientific interest [5].

Recently, Amanna and Slifka reviewed six plausible models describing the evolution of the humoral immune response over time [2]. Four of these models were based on a memory B-cell dependent process, assuming antibody production either due to chronic or repeated infections, persisting antigen immune complexes on the surface of follicular dendritic cells, or cross-reactive antigen stimulation [6], [7], [8], [9]. According to the authors, none of these models is suitable to reproduce the evolution of antibody levels with time after exposure to viral or vaccine antigens. In contrast with the previous approaches, Amanna and Slifka [2] proposed two theoretical models considering plasma cells as an independent B-cell subpopulation that is long-lived even in the absence of replenishment by memory B-cells [5], [10]: the ‘*plasma cell niche competition model*’ and the ‘*plasma cell imprinted lifespan model*’ [2]. There is strong evidence that plasma cells can be long-lived when located in survival niches, especially in bone marrow and to a lesser extent the spleen. These antibody-secreting cells could be pivotal for the maintenance of humoral immunity [11], [12], [13], [14]. As suggested by Radbruch *et al.* [13], the first model was based on the assumption that there is competition between resident and new migratory plasma cells for a finite number of survival niches. New migratory plasma cells are unable to survive for long periods outside of these niches. Since plasma cells accumulate in these niches due to new infections and reinfections over time, the average age of plasma cells occupying the niches increases. Consequently, the duration of the humoral response they induce should decay more rapidly with time. The latter effect remains to be demonstrated [5]. The last model proposed by Amanna and Slifka assumed an “*imprinted*” lifespan for antigen-specific plasma cells [2]. This model explicitly assumed no further division of plasma cells. In the absence of replenishment of memory B-cells (due to reinfection or vaccine boosting), this implies that serum antibody titers would be strongly related to the lifespan of antigen-specific plasma cell populations. Hence, the antibody kinetics can be assumed to evolve over three time-scales: the antibody lifespan, with an half-life ranging between 17.5 and 26 days, the short-lived plasma cell and long-lived plasma cell lifespans. However, as noted by the authors, the imprinted lifespan model does not differentiate between short lived-plasma cells and memory B-cell dependent mechanisms, such as the role of persisting antigen stimulation in the early antibody kinetics, but provides insights on the long-term persistence of antibodies after infection or vaccination and the interplay between antibody titers and plasma cell kinetics. Although based on evidenced immunological concepts, to our knowledge, Amanna and Slifka's models were not used to analyze data and remained purely theoretical.

Several mathematical models have been developed to study the long-term persistence of vaccine-induced antibodies from serological follow-up surveys, using either the general mean titer (GMT) or individual antibody titers as an outcome measure. Most of these studies estimated the decay rate of antibodies assuming a simple exponential decay or including rapid and slow components for decay depending on the time after vaccination. Using these frameworks, long-term persistence (over 25 years) of hepatitis A (HAV) vaccine-induced immunity was demonstrated [15], [16], [17], [18], [19]. Fraser *et al*. [20] proposed a model accounting explicitly for B-cell population (antibody secreting cells) kinetics and extended their model by differentiating an “activated” and a memory B-cell subpopulation [20], [21]. In the present study, a mathematical formulation of the “*plasma-cell imprinted lifespan*” model proposed by Amanna and Slifka [2] was implemented and used to estimate long-term persistence of anti-HAV antibodies from two 10-year follow-up studies in adults vaccinated with inactivated hepatitis A vaccines.

## Materials and Methods

### Data

Two long-term follow-up datasets were used for parameter estimation. Healthy HAV-seronegative adults aged between 18 and 40 years were enrolled after giving their written informed consent [17]. The first dataset included 289 subjects vaccinated with 2 doses of Havrix™ 1440 with 0-6 (109 individuals) or 0–12 months (180 individuals) vaccination schedules. This inactivated hepatitis A vaccine, manufactured by SmithKline Beecham Biologicals and introduced in 1994, was formulated to contain no less than 1440 ELISA units (El.U) of hepatitis A antigen (strain HM175) per 1 ml dose, adsorbed onto 0.5 mg of aluminium salts. Subjects received the vaccine in the right deltoid muscle. Various vaccination schedules were shown to provide similar immune responses [22]. Blood samples were taken in each participant before vaccination, to ensure seronegativity, as well as between the primary and boosting doses, and after booster administration. In view of our aim with the present study - the evaluation of long-term persistence of antibodies after a full vaccination schedule, the dataset we use here is limited to time-points after boosting, *i.e.* at 1, 12, 18, 24, 30, 36, 42, 48, 50, 66, 78, 90, 102, 114 and 126 months after boosting. The second dataset included 113 subjects vaccinated with 3 doses of Havrix™ 720 according to a 0-, 1-, 6-vaccination schedule [16], [23]. This vaccine, which is the predecessor formulation of Havrix™ 1440, contained no less than 720 Elisa units per 1.0-ml dose. Blood samples were taken at 1, 6, 12, 18, 30, 42, 54, 66, 78, 90, 102 and 114 months after the booster dose (6 months). Antibody titration was performed using an “in-house” ELISA inhibition assay [24]. Subjects with antibody levels below 20 mIU/ml for the ELISA test were considered seronegative.

### Mathematical models of antibody kinetics

The “*plasma-cell imprinted lifespan*” model accounting for the dynamics of plasma cell (P) and antibody (A) populations was considered. The plasma cell population is divided in two subpopulations according to their specific lifespan: short- and long-lived plasma cells denoted by 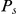 and 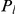, respectively. Assuming no renewal, plasma cell populations decline over time with different decay rates according to their longevity. However, long-lived plasma cells can survive for long periods of time residing in survival niches, mainly in the bone marrow, and could consequently be considered as virtually steady [2], [13], [14]. Finally, assuming that the antibody lifespan is short relatively to plasma cell lifespan, antibody kinetics can be considered to reflect the underlying kinetics of plasma cell populations [2]. Owing to these different assumptions, three nested models were explored.

#### Complete model

The dynamics of plasma cell and antibody populations are described by the following system of differential equations:
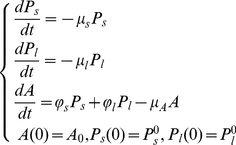
(1)Where 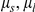 and 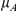 represent the average decay rates of short-lived plasma cells, long-lived plasma cells and antibodies, respectively; 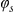 and 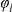 are the production rates of antibodies by short- and long-lived plasma cells, 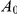 is the initial antibody level, 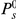 and 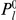 are the initial population sizes of short- and long-lived plasma cells.

This system has the following analytical solution:
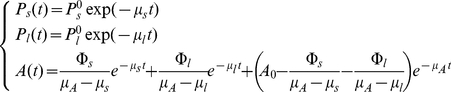
(2)where 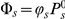 and 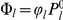


#### Asymptotic model

Assuming that the lifespan of long-lived plasma cells is infinity, *i.e.* 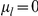, the asymptotic total antibody production rate is a constant different from zero 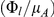. Solution (2) then becomes
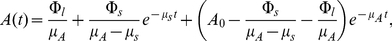
(3)


#### Plasma cell driven kinetic (PCDK) model

Assuming that the antibody lifespan is short relatively to plasma cell lifespan 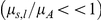, the antibody kinetics can be considered as being an immediate reflection of the underlying kinetics of plasma cell populations [2]. Solution (2) amounts then to
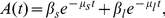
(4)where 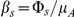 and 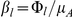.

### Parameter estimation

A non-linear mixed effects model was used to estimate model parameters 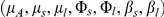
as described by Snoeck *et al.* [25]. Briefly, individual parameters are assumed to be log-normally distributed and were used to predict the antibody titer in an individual 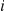
at a certain point in time 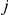 (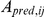) [25]. The measured antibody-titers (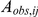) were log10-transformed for the analysis with an additive residual-error:
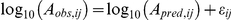



The 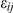 values are assumed to be normally distributed with mean zero and variance 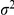. Population parameters were estimated using MLE by the SAEM algorithm for the hierarchical nonlinear mixed-effects model analysis using Monolix software (http://www.monolix.org) [26].

A nonparametric bootstrap procedure was used to determine the 95% confidence intervals of parameter estimates permitting the evaluation of the accuracy of parameter estimates. One thousand bootstrap replicates were generated by resampling individual profiles for each dataset. For each bootstrap replicate, each model was refitted to get an estimate of the population parameters. The 95% confidence interval was constructed from the 2.5^th^ and 97.5^th^ percentiles for each of the population parameters [27]. For each bootstrap replicate, long-term extrapolations of antibody decay were obtained, resulting in predictions and 95% confidence intervals of the mean duration of vaccine-induced immunity (antibody titers higher than 20mUI/ml), as well as the mean time for the proportion of immune individuals to decrease down to 95% and 90%.

### Alternative modeling assumptions: the power-law models

Fraser et al. [20] proposed an alternative to exponential distributions of decay rates, assuming an heterogeneity in the decay rate of B-cells expressed by a gamma distribution. This hypothesis led to the formulation of the so-called “conventional power-law” model previously used to model antibody persistence [28], . In [20], this model was further improved to account for two B-cell subpopulations leading to an “asymptotic model”, assuming that a proportion of the B-cell population does not decrease, and a “full model”, assuming a slower decay rate for a proportion of the B-cell population. Using the notations in [20], these three models describing the antibody kinetics are given by:

1. Conventional power-law model
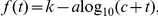

2. Asymptotic power-law model
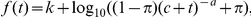

3. Full power-law model
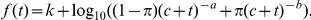

where 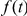 is the 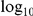-transform of antibody titer at time 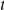, 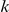
is the peak 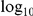-level, 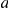 and 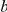 represent the decay rates of short-lived and long-lived plasma cells, respectively, and 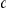 is an arbitrary constant (often set to 0). Finally, 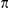 (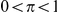) is the relative level of antibodies produced in the long-term plateau. Using the same methodology as previously described, parameters were estimated for each power-law model.

### Model diagnostic

AIC (Akaike Information Criterion) was used for model selection. As population based diagnostics were not very informative, goodness of fit was assessed based on diagnostic plots for the individual predictions (IPRED), and individual weighted residuals (IWRES) by calculation of the ε-shrinkage [31].

## Results

Parameter estimates are given in Table 1. For the complete model, the population average antibody decay rates were close to 0.8 for both datasets (95% confidence intervals [0.63, 1.34] and [0.65, 1.36] for the first and second datasets, respectively), corresponding to a half-life of 26 days. Under the assumption of the asymptotic model, the average decay rate obtained with the first dataset (0.75 [0.49, 1.10]) was slightly lower than the one obtained with the second dataset (0.95 [0.68, 1.48]); these values remained consistent with the literature (half-lives of 27.7 and 21.9 days, respectively) [2]. Using the individual estimates of the decay rate parameter provided by Monolix as the mean of their posterior distribution [26], we performed Kruskal-Wallis tests to investigate the difference between the kinetics at early time-points after the boosting dose according to model assumptions (complete and asymptotic) and vaccine formulation (Havrix™ 1440 and Havrix™ 720). Although no difference was found between the two models (p = 0.84), a significant difference was shown between the two vaccines, with a higher decay rate for the oldest vaccine (Havrix™ 720; p = 0.004). However, this difference could also be due to the inclusion of the 6-month time-point in the second dataset which allows for a decomposition of the kinetics according to the different population time-scales. Moreover, under the asymptotic assumption (lowest AIC), the inter-individual variability, estimated as the standard deviation of random-effects [26], was reduced from 84% (Havrix™ 1440) with the first dataset to 61% for the second dataset (Havrix™ 720). Note that when looking at the exclusion of random effects one by one, all were significant (5% significance level) based on a 50∶50 mixture of a 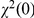 and 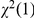 distribution.

**Table 1:** Parameter estimates according to the modeling assumptions: complete, asymptotic or plasma-cell driven kinetics (PCDK) model (95% confidence intervals determined using bootstrap percentile intervals).

	Population parameter estimates (CI)					
	Havrix™ 1440 dataset			Havrix™ 720 dataset		
Parameters	Complete Model	Asymptotic Model	PCDK Model	Complete Model	Asymptotic Model	PCDK Model
Φ*_s_* (1e^3^ mIU/ml* Month^−1^)	1.12 (0.81, 2.20)	1.04 (0.55, 1.71)	-	1.00 (0.65, 1.37)	0.97 (0.68, 1.72)	-
Φ*_l_* (1e^3^ mIU/ml* Month^−1^)	0.54 (0.43, 0.92)	0.51 (0.33, 0.75)	-	0.26 (0.20, 0.59)	0.40 (0.20, 0.65)	-
*β_s_* (1e^3^ mIU/ml)	-	-	3.38 (2.95, 3.96)	-	-	5.56 (3.89, 8.01)
*β_l_* (1e^3^ mIU/ml)	-	-	0.84 (0.70, 0.97)	-	-	1.43 (1.15, 1.71)
*μ_s_* (Month^−1^)	0.069 (0.062, 0.080)	0.07 (0.058, 0.074)	0.14 (0.12, 0.16)	0.014 (0.011, 0.026)	0.02 (0.013, 0.028)	0.76 (0.51, 1.04)
*μ_l_* (Month^−1^)	1.8e^−6^ (5.2e-7, 7.8e-6)	-	1.5e^−3^ (3.03e-5, 2.3e^−3^)	9.8e^−4^ (1.4e^−4^, 1.3e^−3^)	-	8.1e^−3^ (6.1e^−3^, 9.8e^−3^)
*μ_A_* (Month^−1^)	0.79 (0.63, 1.34)	0.75 (0.49, 1.10)	-	0.82 (0.65, 1.36)	0.95(0.68, 1.48)	-
*A_0_* (1e^3^ mIU/ml)	7.79 (6.38, 12.21)	7.60 (5.90, 10.66)	-	8.62 (6.32, 14.6)	9.26 (6.27, 15.41)	-
*AIC*	−1626.63	−1630.63	−1354.10	−346.2	−346.35	−308.16
*ε*-shrinkage (%)	16	16	13	18	17	13

Testing whether 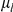 is significantly different from 0 was done using a likelihood ratio test for which the asymptotic null distribution is a 50∶50 mixture of a 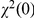 and 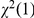 distribution [32], [33]. The estimate of 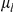 was not found to be significantly different from 0 with the complete model, meaning that the lifespan of long-lived plasma cells cannot be estimated and this subpopulation could be considered constant. Nevertheless, the inclusion of a supplementary data-point in the early stage of the kinetics (6 month post-boosting; Havrix™ 720 dataset) permitted to improve the estimation accuracy for the long-lived plasma-cells decay rate, decreasing substantially the relative standard error (RSE) of the estimate from 2e^4^% for the first dataset to 231% for the second (data not shown). Discarding the additional 6-month data point from the second dataset, the asymptotic model resulted in estimates of the antibody decay rate close to the one obtained with the first dataset (data not shown). This result suggests that more time points during the first year would allow estimating the three time scales using the complete model. The third model (PCDK) assumed that the antibody decay rate can be ignored relative to the plasma-cell kinetics, leading to an “adiabatic” formulation. For both datasets, the time scales obtained for the short- and long-lived plasma cell lifespan differ by two orders of magnitude. For the first dataset (Havrix™ 1440), the estimated lifespan of short-lived plasma cells (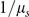), averaged around 7 months, which is much longer than the 1 month antibody lifespan. The estimated lifespan of long-lived plasma cells, averaged around 60 years (i.e. roughly similar to the average human lifespan). For the second dataset (Havrix™ 720), the estimated lifespan of short-lived plasma cells was close to 1 months and the estimated lifespan of long-lived plasma cells was only 10 years. However, due to the additional measurement at 6 months after the (final) booster dose the adiabatic assumption is no longer valid (ignoring the antibody lifespan compared to the plasma cell lifespan). Indeed, at the 6 months post booster point, the observed antibody kinetics are principally driven by the antibody decay rate, implying that we can no longer assume that its effect is negligible relative to that of the plasma-cell kinetics. In both cases, the estimates of 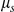 are the result of a combination of antibody and short-lived plasma cell decays. However, the lifespan of long-lived plasma cells, contributing to long-term persistence of the humoral response, was found to be 6-fold longer with the more recent and more potent vaccine formulation (Havrix™ 1440) than with the older formulation (Havrix™ 720). The conventional power-law model assumes that the antibody level declines continuously with time but the data suggest the existence of at least two phases of decline: a short-term component with a high decay rate in the first 2 years of observation, followed by a long-term component which could be thought as a “plateau” phase. The results obtained for the two datasets using the conventional power-law model are similar with a low decay rate (a = 0.63) reflecting both phases using only one parameter (Table 2). The inclusion of an asymptotic phase in the modified power-law model allows for a focus on the short term dynamics. For both datasets, the decay rate estimates were drastically increased compared with conventional power-law approaches. The decay rate obtained with the second dataset was slightly lower than for the first dataset, but combined with a lower peak of the antibody titer, the immunity provided by the Havrix™ 720 vaccine remains weakest compared to the more recent Havrix™ 1440 vaccine. Finally, the introduction of the second time scale, governing the long-term behaviour, referred as “full power-law model” supports the results obtained in our study: the presence of a supplementary point (6 months post-boosting) in the second dataset allow for a better estimation of the long-term component. The results obtained with the first dataset are close to the ones obtained with the asymptotic model with a decay rate close to 0 (b = 0.07) whereas the second data set permitted to estimate a decay rate of 0.37 for long-lived plasma cells resulting in a slow but continuous decay of the antibody population.

**Table 2:** Parameter estimates using power-law model (95% confidence intervals determined using bootstrap percentile intervals).

	Population parameter estimates (CI)					
	Havrix™ 1440 dataset			Havrix™ 720 dataset		
Parameters	Conventional power-law	Asymptotic power-law	Full power-law	Conventional power-law	Asymptotic power-law	Full power-law
*k*	4.13 (4.04, 4.18)	5.87 (5.67, 6.12)	6.21 (5.65, 6.97)	4.00 (3.89, 4.10)	5.29 (4.48, 5.74)	6.37 (6.12, 6.55)
*a*	0.63 (0.59, 0.67)	2.26 (2.07, 2.50)	2.79 (2.09, 3.40)	0.60 (0.54, 0.66)	2.01 (0.93,2.48)	3.67 (3.28, 3.88)
*π*	-	8.1e^−4^ (4.3e^−4^, 1.2e^−3^)	0.0008 (1.8e^−4^, 1.4e^−3^)	-	3.2e^−3^ (1.3e^−3^, 5.1e^−3^)	1.7e-^3^ (9.7e^−4^, 2.8e^−3^)
*b*	-	-	0.08 (1.8e^−3^, 0.16)	-	-	0.37 (0.29, 0.43)
*AIC*	−572.83	−1226.77	−1255.01	−128.36	−204.26	−297.35

**Figure 1:**
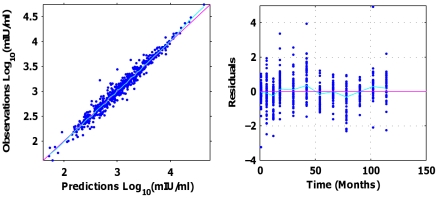
Observations *Vs*. model predictions (left) and residuals Vs Time (right) plots using individual parameters (Havrix™ 720 dataset, Asymptotic model, log_10_ scale).

All models showed a good consistency between individual predictions and observations with 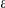-shrinkage estimated between 13 and 18%. Additional data points in the early phase of the kinetics might decrease the 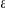-shrinkage as they provide more information on high-level antibodies. Among the six models considered throughout this study, the lowest AIC was obtained with the asymptotic model assuming exponential decays for antibodies and plasma cells. This model is a derivation of the complete model by constraining the decay rate of long-lived plasma cells to 0. Figure 1 displays the observation/prediction plot (log_10_ scale) for the asymptotic model (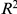 = 0.97).

**Figure 2:**
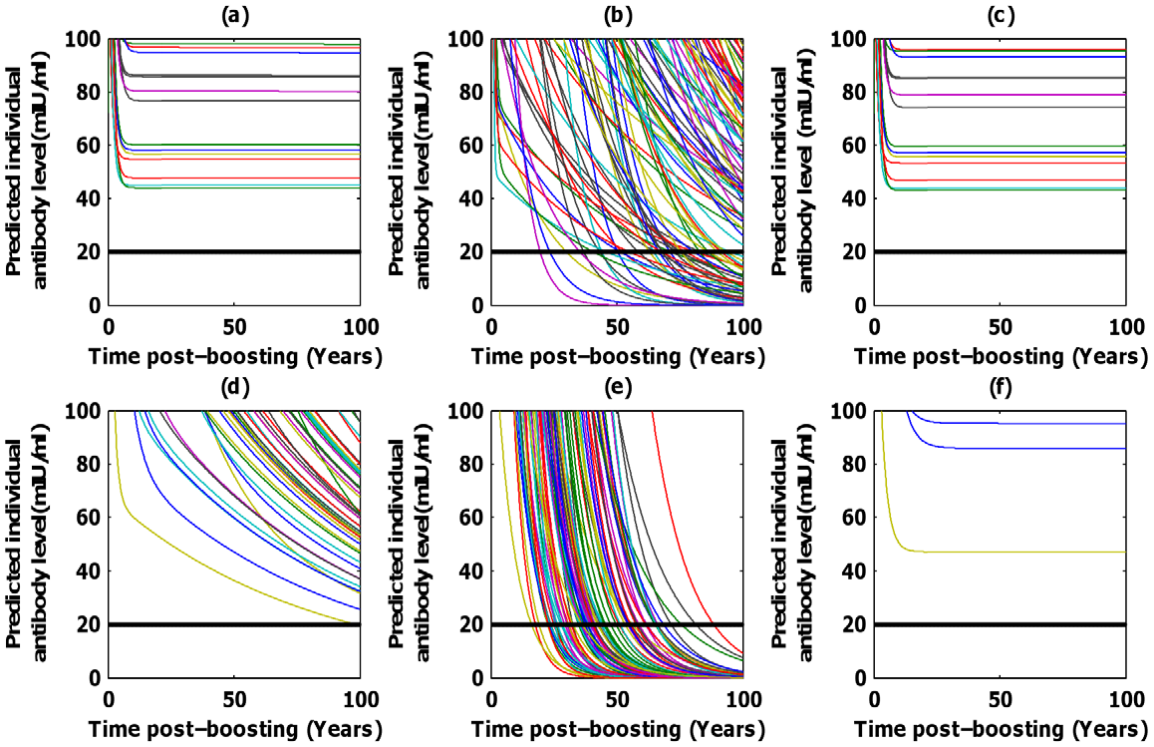
Individual prediction plots with a focus around the positivity threshold (20 mIU/ml, black line). (a,c,b) Havrix™ 1440 dataset, (d,e,f) Havrix™ 720 dataset; (a,d) complete model, (b,e) plasma-cell driven kinetics model, (c,f) asymptotic model.

**Figure 3:**
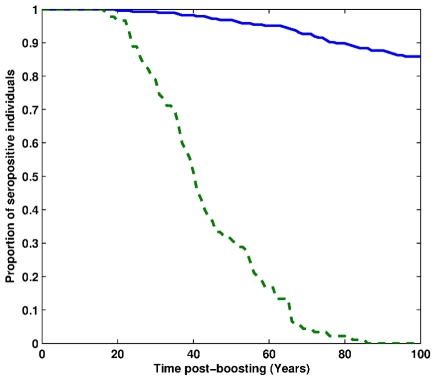
Predicted proportion of seropositive patients according to time post vaccination from the plasma-cell driven kinetics model (full blue line: Havrix™ 1440 dataset , dashed green line: Havrix™ 720 dataset).

Although care has to be taken using these models based on 10 years of data, long-term individual extrapolations of antibody kinetics were derived from the individual empirical parameter estimates for each model (complete, adiabatic and asymptotic) and the two data sets (Figure 2). In accordance with international current practice, the positivity threshold was fixed to 20 mIU/ml and subjects with antibody levels below this threshold for the ELISA test were considered seronegative. Immunity was considered as lost when a subject passed from seropositive to seronegative status [24], [34]. A focus around the positivity threshold (20 mIU/ml, thick black line) was plotted for each model and dataset to monitor the population serological response according to time post-boosting. For the first dataset, including only one point in the first year after vaccination (1 month), the asymptotic, complete and power-law models gave similar results with a life-long immunity for all vaccinated patients. Conversely, for the adiabatic PCDK model a proportion of the population loses humoral immunity, with the first seronegative patient occuring 20 years after vaccination. However, the proportion of seronegative patients 100 years after vaccination did not exceed 15% (figure 3), showing a good long-term efficacy of the vaccine. The mean time to immunity waning was 216 years (95%confidence interval [143.0, 848.6], table 3). The results for the second data set differ according to the model assumptions. Although the asymptotic model gave similar results as for the first dataset predicting lifelong immunity due to the supposed asymptot, results with the complete and adiabatic approach were divergent. The complete model was found closer to the adiabatic due to the existence of an additional sample time in the early phase of the kinetics (6 months). Although the power-law models predicted lifelong immunity for both vaccines, the estimate of the decay rate of long lived plasma-cells was found to be higher for the second dataset, confirming that the “plateau” assumption in the asymptotic model provides crude approximations of the actual long-term kinetics. Adiabatic model predictions showed that the total population lost immunity within 100 years after vaccination. Moreover, the mean time to lose immunity was evaluated to be 43 years (95% confidence interval [34.8, 52.0]; Table 3).

**Table 3:** Long-term prediction of HAV antibody dynamics obtained with complete and plasma cell driven kinetics (PCDK) models (95% confidence intervals determined using bootstrap percentile intervals).

	Havrix™ 1440 dataset		Havrix™ 720 dataset	
	Complete Model	PCDK Model	Complete Model	PCDK Model
Mean Time to immunity waning (years)	1.7e^5^ (4.7e^4^, 6.7e^6^)	216.1 (143.0, 848.6)	237.1 (188.5, 1.7e^3^)	43 (34.8, 52.0)
Time below 95% of immune patients (years)	7.6e^4^ (1.7e^4^, 3.4e^5^)	63 (31.6, 576.9)	147.1 (111.2, 1.1e^3^)	23.4 (17.7, 25.3)
Time below 90% of immune patients (years)	1.0e^5^ (2.8e^4^, 4.3e^5^)	77.4 (52.6, 681.4)	169.4 (126.6, 1.2e^3^)	24.4 (22.2, 29.3)

## Discussion

A mathematical model, based on the “*imprinted plasma cell lifespan model”* proposed by Amanna and Slifka, was developed to study the long-term persistence of antibodies after vaccination with inactivated HAV vaccines [2]. Previous studies showed that anti-HAV antibodies can persist for at least 25 years and that a two-phase decay of antibody levels occurs according to the time since vaccination [35], [36]. However, the models used for the estimations were solely based on the antibody dynamics and did not handle the underlying immunological mechanisms. Plasma-cells are the main antibody-secreting cells and it is currently recognized that some of these cells can survive for extended periods when located in survival niches, especially in the bone marrow [12], [13], [14]. The model used in our study assumed that the antibody kinetics are determined by three time-scales: the antibody, the short-lived plasma cell and long-lived plasma cell lifespans (complete model). Two other approaches were derived from the complete model:

- assuming a constant long-lived plasma cell population (asymptotic model) close to the model of Fraser *et al*. [20].
- ignoring the antibody lifespan (assumed to be short compared with plasma-cell lifespans (plasma cell driven kinetic model)).

The complete model, which should be the best representation of the actual process including three time-scales (antibody, long- and short-lived cell life-spans), did not allow for accurate estimates, especially concerning the decay rate of long-lived plasma cells (RSE>200%). The asymptotic model permits to estimate the antibody decay rate corresponding to the shortest time scale (around 1 month) [3]. However the hypothesis of the asymptotic model, assuming a constant antibody production by long-lived plasma cells residing in niches in the bone marrow and considered as surviving in the host for life, generates a cost on long-term predictions of the antibody decay which cannot be studied using this approach. The third approach, called “plasma cell driven kinetic”, considers the antibody kinetics to immediately reflect the underlying kinetics of plasma cell populations. Thus, ignoring the antibody decay, which cannot be distinguished from plasma-cells, allows for fitting the long-term kinetics. However, the interpretation of the parameters is not straightforward, especially when detailed data are available in the initial phase of the kinetics, which corresponds to the antibody decay (table 2). Although our model selection criterion (AIC) tends to select the asymptotic model, all three models have their own interest depending on the research question:

1. Asymptotic model: Study of the short-term antibody decay and particularly the duration of antibody lifespan.
2. Plasma cell driven kinetic model: Study of long-term behavior, permitting to estimate the mean time to waned immunity.
3. Complete model: Global approach that could allow dealing with the two previous research questions. However, this approach would need additional data, especially in the initial phase of the antibody decay after vaccination, which would permit to identify the transition between the adiabatic and the asymptotic hypotheses.

Combining the results obtained with each of these models, the average antibody lifespan was estimated to be around one month that is consistent with the literature whereas the average plasma cell lifespans varied from 3 to 7 months for short-lived plasma-cells, and over 60 years for long-lived plasma cell.

Power-law models present a relevant alternative to the modelling framework based on plasma cells imprinted lifespan, both from a methodological and from a biological point of view. In absence of emperical evidence for “heterogeneity in the decay rate of B-cells” given the data at our disposal, exponential decays were assumed for short-lived and long-lived plasma-cells. The main results of our study rely on the fact that three time-scales were biologically relevant to explain the antibody decay: the antibody, the short-lived and long-lived plasma cell lifespans. The power-law models as described in Fraser et al. [20] included at most two time-scales, which could explain the differences observed in the fits. This conclusion is supported by the results obtained with the “Plasma-Cell Driven Kinetics” (PCDK) model, which accounted for two time-scales and for which the AIC values were close to the one obtained with the full power-law model (also accounting for two time scales). Thus, whenever relevant data would be available, the coupling of the two approaches offers an appealing perspective for future immunological research.

Using individual parameter estimates, the mean time to immunity waning was estimated to be 43 years for the individuals vaccinated with Havrix™ 720 vaccine. Similar results were previously obtained by Van Herck *et al.* [16] who estimated the individual slow decay rate of antibodies (between months 76 and 128 post boosting) and estimated the mean number of years before an individual reached the seroconversion level (20mIU/ml) to 45 years. With the same methodology, less than 15% of individuals vaccinated with the latest vaccine formulation (Havrix™ 1440) were estimated to lose their immunity 100 years after boosting, showing possible life-long vaccine-induced immunity. Although these results are based on long-term extrapolation and could be influenced by immunosenescence and other distortions of immunity, they elucidate in a simple way the observed differences between the two vaccines.

Accounting for correlations between random effects was not found to impact the accuracy of parameter estimates obtained with the PCDK model (data not shown). Computational problems, due to convergence failure, avoided the inclusion of such correlations when analyzing the data with the asymptotic and complete models. However, based on the results obtained with the PCDK model, the main conclusions of this study are deemed to be robust to this specific misspecification of the random effects distribution. The effect of such misspecification would require further research which is beyond the scope of the present study.

These results have a number of direct implications:

- In immunology, it offers a quantitative assessment of the time scales over which plasma cells and antibodies live and interact. This insight may provide a basis for further quantitative research on the immunology, with direct consequences for understanding the epidemiology of infectious diseases.
- In vaccinology, it offers an opportunity for clinical trial researchers to collect relevant information early on, in order to make long term predictions on immunity conferred by vaccines. We showed in particular that antibody levels measured within a year after a booster dose provide highly relevant information for long term predictions of protective immunity over time.
- In health policy, it offers more than a purely intuitive basis to make recommendations on booster vaccinations. Our models for hepatitis A suggest that this would not be required at least within a 40 year time span after the booster vaccine dose.

A further improvement of our mathematical model could include the explicit interaction between humoral and cellular immunity. This would involve nonlinear coupling terms. The validation of such theoretical generalisations would require much more refined data not only about antibodies but also about B-cell and T-cell subpopulations.
